# A Pragmatic Internet Intervention to Promote Positive Parenting and School Readiness in Early Childhood: Initial Evidence of Program Use and Satisfaction

**DOI:** 10.2196/14518

**Published:** 2019-11-29

**Authors:** Lucy McGoron, Hilary Horn Ratner, Kathryn AG Knoff, Erica Hvizdos, Steven J Ondersma

**Affiliations:** 1 Wayne State University The Merrill Palmer Skillman Institute for Child and Family Development Detroit, MI United States

**Keywords:** child rearing, child development

## Abstract

**Background:**

Internet-based parenting programs have the potential to connect families to research-informed materials to promote positive child development. However, such programs can only succeed to the extent that the intended population engages with them.

**Objective:**

This study aimed to evaluate engagement in the 5-a-Day Parenting program, a technology-based program designed with low-income families in mind, to promote daily use of 5 specific parenting activities conducive to children’s school readiness. Following earlier pilot data, the program was enhanced with an initial motivational e-intervention and tailored text messages designed to promote engagement.

**Methods:**

Parents were recruited from local childcare centers and through a participant registry. We examined rates of receipt of program text messages and use of video-based content on the program website, 3 factors that may affect website use, and satisfaction with key program elements.

**Results:**

A total of 360 parents of young children learned about the study and had the opportunity to use the 5-a-Day Parenting website. Of these, 94 parents participated in the study, and 33% (31/94) accessed the video-based content on the website at least once. No association was found between website use and program recruitment approach, program-affiliation message, sociocontextual risk, and baseline use of the five parenting activities. Satisfaction with text messages and video-based content was high.

**Conclusions:**

For some parents, technology-based programs appear useful; however, engagement could still be enhanced. Additional research should seek innovative strategies for promoting engagement in Web-based parenting programs.

## Introduction

### Background

Young children’s successful entry into school is closely associated with their social, emotional, cognitive, and academic readiness [1], each of which is strongly influenced by parenting and the home environment. For instance, parental warmth promotes positive emotional development [2,3] and reduces children’s behavioral problems [4]. Cognitively stimulating home environments, conducive to language and book sharing, enhance children’s language development and early reading skills [5]. Sensitive parent-child play builds social competence [6]. Even the overall structure of children’s days is important; children from families that have regular routines, share meals together, and follow a bedtime routine have stronger emotion regulation and social skills [5,7,8]. Unfortunately, children in families experiencing heightened sociocontextual risk (eg, financial struggles) are more likely to have deficits in school readiness skills [5], in part, because high levels of sociocontextual risk creates stress for parents, which in turn can interfere with positive parenting practices [9].

Face-to-face parenting programs teach parenting strategies that promote children’s school readiness skills (eg, Landry et al [10]) and reduce challenging behaviors (eg, Kaminski et al [11] and Webster-Stratton et al [12]) that can interfere with school readiness. However, McGoron and Ondersma [13] identify a number of barriers to the use and completion of such programs. Lack of access to services, practical barriers (eg, lack of transportation), stigma around seeking parenting advice, lack of information about where to find services, family stressors, and a simple lack of interest in services all serve to inhibit engagement and continued use in potentially helpful parenting programs. Internet-based delivery of parenting programs may ameliorate many of the above-noted barriers [13,14]. As is often noted, internet access is becoming ubiquitous, even among parents facing sociocontextual risk [15].

With this knowledge, we created the 5-a-Day Parenting program, a fully technology-based program, to promote school readiness in early childhood through positive parenting. Development of this program was influenced by the domain-specific approach to socialization proposed and outlined by Grusec and Davidov [16]. This approach integrates multiple theories of child socialization (eg, attachment theory, social learning theory), recognizing that there are distinct domains of parenting behavior (eg, protection, reciprocity, control and guided learning) that are related to specific child outcomes. The 5-a-Day Parenting program taps into multiple socialization domains given that the outcome of focus is school readiness, which is multifaceted and includes children’s development of social, emotional, cognitive, and behavioral skills. Ultimately, the goal of the program is to distill a large body of child development research (eg, [1-8]) into 5 specific parenting behaviors. The 5 behaviors include (1) reading at least one book a day to children; (2) playing with children at least 10 min a day; (3) sharing at least one meal a day; (4) showing affection each day; and (5) following a bedtime routine. Multiple domains of optimal parenting are targeted within these specific activities. For instance, parents can learn about optimal guided learning (eg, labeling and scaffolding) while playing with their children, reading to their children, and sharing a meal.

**Figure 1:**
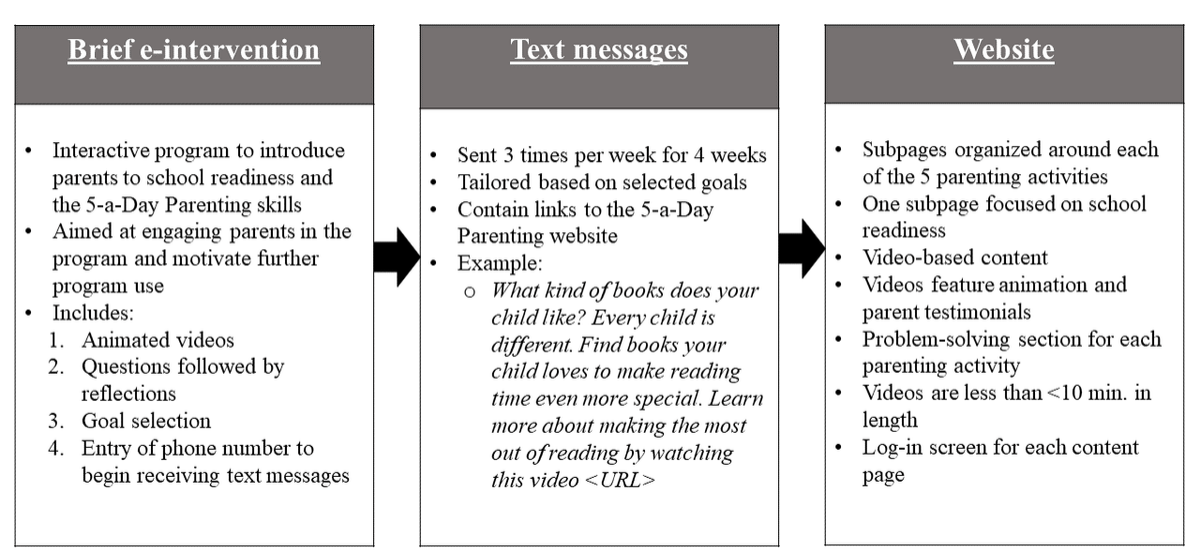
Overview of the 5-a-Day Parenting program.

The program is intended to be highly practical rather than intensive to keep in mind the demands faced by busy parents who may be experiencing multiple sociocontextual stressors. As such, the program was created with low-income families in mind because these families face barriers to attending the face-to-face parenting program [17,18]. The 5-a-Day Parenting program website teaches parents about the benefits of the 5 activities, how to make the most out of time spent doing these activities, and how to overcome related challenges. At the beginning, parents engage with the program by selecting which of the 5 activities to focus on. Figure 1 outlines steps in the 3-part program.

The rate at which parents will use internet-based parenting programs in general, and the 5-a-Day Parenting program specifically, is still unclear. Current investigations of internet-based parenting programs often describe the final sample of participating parents but do not indicate what percentage of parents declined (eg, [19,20]), but there are several exceptions. In a sample of military parents, Doty et al [21] found about half (193/370) of the participating families used a Web-based parenting resource; however, more than 70% (271/370) attended a face-to-face parenting program session. For parents of children with attention-deficit/hyperactivity disorder (ADHD), Ryan et al [22] reported that nearly 60% (91/158) of parents used an educational website. Investigations with these special populations, however, provide little insight into the use of a parenting website for a *general* population of parents.

Specific to the 5-a-Day Parenting program, our initial pilot investigation [23] demonstrated that while most parents reported intentions to use the website, actual traffic to the website was low, and some parents reported needing reminders. Learning from this process, we made program enhancements to promote program engagement, including adding a brief e-intervention at initial engagement to motivate program use and text messages to reinforce content and provide reminders. Although these new program features may promote engagement, other factors may also affect parents’ use of the 5-a-Day Parenting website. Unfortunately, there is little research to suggest which factors may affect parents’ use of an online program. We selected 4 possible factors to explore. First, where or how parents learn about an online parenting program may affect use of the 5-a-Day Parenting website. For example, parents may be more open to using a program if they learn about it face-to-face from a trusted source (eg, a service they already use, such as childcare or a pediatric practice). Second, stated program affiliation may also affect the use of the 5-a-Day Parenting website. Consistent with this idea, the qualitative work of Bernhardt and Felter [24] found that parents rated Web-based resources created by experts or academics as more trustworthy. Moreover, Eysenbach and Kohler [25] also reported that consumers look for online health information that appears scientific. Thus, programs with an academic or scientific affiliation may engender more program use. Third, we also considered whether the level of sociocontextual risk affected use of the program. Baker et al [26] reported that low-risk and high-risk families were equally open to using an internet-based parenting program. However, sociocontextual risk, such as economic strain, creates stress and daily hassles for parents (see Masarik and Conger [27], for a review), which could inhibit the use of an online program. Thus, it is important to consider the possible impact of sociocontextual risk on *actual* program use. Finally, given that parents who already regularly engage in the five parenting activities may be less inclined to use the video-based content on the website, we explored if baseline reports of using the parenting activities were related to website use.

### Objectives

This study had 3 goals. First, we sought to evaluate the use of the 5-a-Day Parenting program; specifically looking at how many parents would continue receiving text messages after going through a brief e-intervention, intended to prompt program use, and how many parents would use the video-based content on the 5-a-Day Parenting website. Second, we sought to evaluate potential factors that may affect use of the video-based content on the program website, including engagement approach (ie, how parents learned about the program), program-affiliation message (ie, stated academic/scientific affiliation or no stated affiliation), and sociocontextual risk. We also report parents’ use of the five parenting strategies at baseline and their relation to use of the video-based content on the program website. Third, we evaluated parents’ satisfaction with the text messages and video-based content on the 5-a-Day Parenting program website.

## Methods

### Participants

Participants were parents of children aged 2 to 5 years in Detroit, Michigan. Participants were recruited from 1 of 2 sources (ie, 2 different recruitment groups). First, parents were recruited through childcare centers that are part of a community-based, university-anchored consortium (referred to hereafter as *the Consortium*; see [28], for details). A total of 6 Consortium childcare centers agreed to allow recruitment for this study. The second recruitment source was a registry consisting of Detroit-area parents of preschool-age children who had provided consent to be contacted about research opportunities.

### Intervention

The 5-a-Day Parenting program is a newly developed program that encourages parents to do 5 daily parenting activities (see [23]). The program also gives parents information about optimizing time together by being responsive and cognitively stimulating during activities (eg, pointing to pictures in books, labeling shapes during play). The program was created by a developmental psychologist (the first author) after reviewing the literature on parenting practices and positive development in young children and identifying specific parenting activities correlated with outcomes important for children’s school readiness. After piloting the program, 2 program enhancements were added. First, we added an initial, brief e-intervention (<10 min) to introduce the five parenting activities, build investment in change, and promote use in the program. This brief e-intervention is interactive, with voice-over narration as well as motivational strategies and goal setting, and includes a video providing information about school readiness and the 5-targeted parenting strategies. Second, we also added a request for the parent’s mobile phone number to enable receipt of tailored text messages. These text messages are sent 3 times per week for 4 weeks, and function as cues to use the video-based content on the 5-a-Day Parenting website. Text messages are tailored based on each participant’s specific goals (eg, reading to their child more often). A link to video-based content in the 5-a-Day Parenting website is included in each text message.

### Recruitment

The Wayne State University Internal Review Board approved all procedures before data collection. Childcare center recruitment involved distribution, at pickup and drop-off times, of folders with information about the 5-a-Day Parenting program and the URL for study participation. The project team tracked rates of folder distribution. Participant registry recruitment involved text messaging parents from the registry with an invitation that included a URL leading to further information about the study. The project team tracked the number of text messages distributed and phone numbers that were no longer in service.

#### Manipulation of Program Affiliation

For parents recruited through Consortium childcare centers, 3 centers were randomly selected to receive a program-affiliation message, and 3 centers were randomly selected to receive a nonaffiliated message. Similarly, participants recruited via the registry were randomly assigned to receive a program-affiliation message or nonaffiliated message.

#### Program-Affiliation Message

In the program-affiliated condition, participants learned that the 5-a-Day Parenting program was developed by Consortium leaders who were experts in child development research. Specifically, the video in the brief e-intervention explicitly stated that the 5-a-Day Parenting program creators were early childhood Consortium leaders (through the University) with expertise in child development and school readiness. In addition, the flier in the folder/text message they received had a link to a subpage on the Consortium website with information about the affiliation; this page is where they began participating.

#### Nonaffiliated Condition

In the nonaffiliated condition, there was no indication that the program was developed by Consortium leaders or by child development experts at a university. The video in the brief e-intervention made no mention of program affiliation. Moreover, the flier in the folder/text message they received had a link to a subpage on the 5-a-Day Parenting website (which had no mention of the university/Consortium) and not a link to Consortium website.

#### Procedures

For all parents, those who chose to participate in the study first went to a URL (provided in their recruitment folder or text message). Participants went through an online study consent, and those providing consent then completed an online survey that took approximately 15 min. Following completion of the survey, parents were led directly to the brief e-intervention, which described the 5-a-Day Parenting program (through a brief video), and allowed them to select goals for change related to the five parenting activities (eg, reading to their child more often [see Figure 1]). Participants recruited from childcare centers received a US $25 Target gift card after completing these baseline participation steps; participants from the participant registry received a US $25 credit on a debit card provided to them as part of registry participation. Immediately following completion of the brief e-intervention, parents received a welcome text (see Figure 1) with a reminder that they could text *STOP* to end text messaging.

Participants were free to use the video-based content on the 5-a-Day Parenting website as much, or as little, as they chose. Parents received no compensation for their use of the website. Parents were required to enter their 3-digit ID number each time they went to a content page (ie, anything beyond the landing page). A total of 4 weeks after baseline participation, parents received a text inviting them to complete an online follow-up survey. The survey asked them to evaluate the text messages they received and the program website. Parents were compensated with a US $25 gift card or US $25 credit on their debit card for their time completing the follow-up survey.

### Measures

#### Demographics

At baseline, parents answered questions about their child’s gender and age. Parents also reported on their age, race, education, relationship status, and perceived financial strain.

#### An Accumulation of Sociocontextual Risk

We created a cumulative risk index to measure sociocontextual risk. Rutter [29] first proposed cumulative risk indices to understand how areas of risk converge to affect children’s adjustment. Importantly, although the areas of risk and number of risk factors included vary across studies, cumulative risk indices in general are related to a number of outcomes from child adjustment [30], parenting [31], and dropout of parenting programs [32]. The creation of a cumulative risk index is straightforward: identify salient risk factors, dichotomize the risk factors (0=no risk; 1=risk), and sum. In all, 4 dichotomized areas of sociocontextual risk, obtained through the baseline questionnaire, were used to create a cumulative risk index. Areas of demographic risk measured were (1) struggling financially (*risk*=responding that they do not *always* have enough money to pay for basic needs), (2) being a single parent (*risk*=not selecting being married or having a romantic partner), (3) low educational attainment (*risk*=reporting no education beyond high school), and (4) and being a young parent (*risk*=being 26 years of age or younger, which was 1 *SD* below the mean for this sample). We selected these areas of risk as they likely create challenges and adversity in parents’ life and may limit program use. Moreover, such areas of risk are generally included in cumulative risk indices (eg, see [33]). The dichotomized risk variables were summed to create a cumulative risk score with a possible range of 0 to 4.

#### Use of Five Parenting Activities Before Participation

At baseline, parents were asked to *think back to how you spent time with (child’s name) over the past week. Rate how often you did the following 5 things*. Parents then went through a list of the five parenting activities and rated how many days they did each the previous week (ranging from 0 to 7 days). In addition to looking at the ratings individually, a total parenting activities score was created by summing responses to the 5 items (possible range of 0 [ie, parents did none of the parenting activities the previous week] to 35 [parents did all 5 of the activities every day in the previous week]).

#### Text Message Use

We recorded the frequency with which parents elected to stop receiving text messages. In addition, in the follow-up survey we asked parents, *How often did you read the text messages you received from the 5-a-Day Parenting Program?* with response options ranging from *Rarely* to *Always*.

#### Website Video-Based Content Use

Project staff tracked log-ins to content pages on the website and connected the log-in ID to the parents’ baseline survey responses, recruitment group, program-affiliation message group, and follow-up survey responses. A binary website use variable (0=no website use; 1=website use) was computed as well as a variable reflecting the number of times each parent logged into the website (frequencies ranged from 0 to 56 times).

#### Evaluation Ratings

The first author wrote the evaluation rating questions for this investigation following the Technology Acceptance Model [34]. A total of 9 items (6 positively worded and 3 negatively worded) focused on text messages. These items elicited parents’ feedback on the helpfulness of the texts in making parenting changes and serving as a reminder to use the website, if parents continued reading the messages, and how much they liked/disliked the messages. A total of 16 items elicited parents’ feedback on the website (11 positively worded and 5 negatively worded). These items asked parents to rate the look and quality of content on the website in general and videos specifically and the amount of information, using response options of *Not at all true*, *Somewhat true*, or *Very true*. In addition, regarding text messages, parents were asked (with yes and no responses) if they would *sign up for text messages if involved in a project like this again?*

#### Statistical Analysis

Before conducting analyses to examine study goals, we examined demographics of the participants. We also examined rates of sociocontextual risk and use of the five parenting activities at baseline; these rates were examined for the whole group and split by participation group. We examined the proportion of participants introduced to the research opportunity who chose to participate in the study at baseline. Next, in line with the first investigation goal, for those that participated, we computed the frequency with which they stopped text messages, their self-reported frequency of reading texts, the frequency of using the website at least once, and the mean and standard deviation of website visits among parents who did use the website. In line with the second investigation goal, paired-samples chi-squared analyses determined if recruitment group or program-affiliation message affected website use. In addition, a bivariate correlation was computed to determine if levels of sociocontextual cumulative risk were associated with website use. A bivariate correlation was also computed to determine if baseline use of the parenting activities was associated with website use. Finally, in line with the third investigation goal, we computed frequencies of responses to parent evaluation ratings of the text messages and website (among those who used the website).

## Results

### Participants

**Figure 2:**
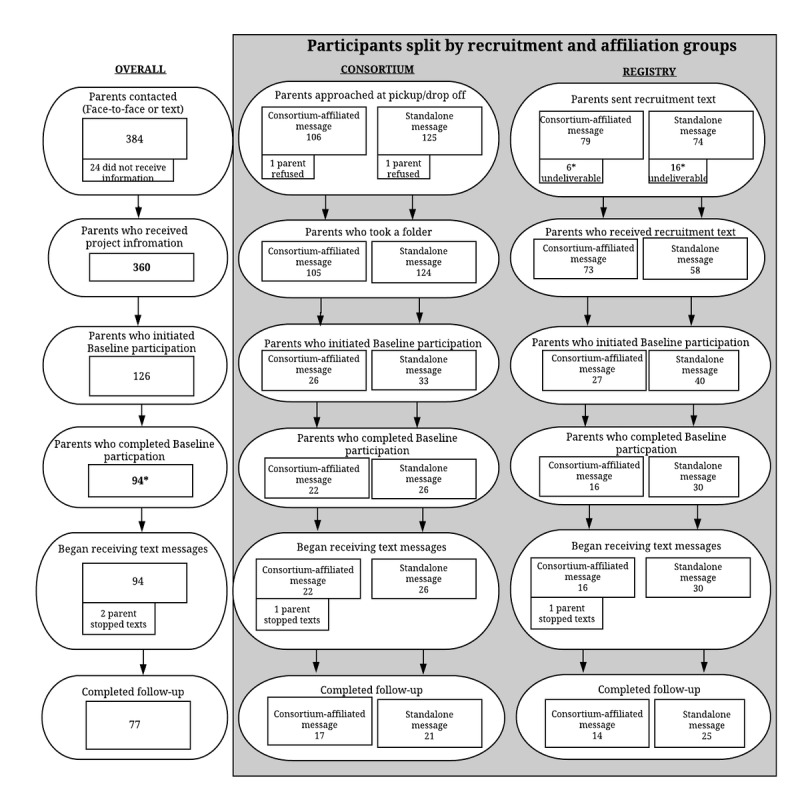
Recruitment and participation summary. *We considered what percent of these parents continued receiving text messages and used the video-based content on the website.

Overall, participant flow is presented in Figure 2. In total, we attempted to inform 384 parents about the opportunity; however, 24 did not receive the information (2 parents refused to take a folder at the childcare center, and 22 phone numbers through the participant registry were nonworking). Thus, 360 parents learned about the 5-a-Day Parenting program by receiving a folder at their Consortium-affiliated childcare center (n=229) or through receiving a text message from the registry (n=131). Of these parents (ie, those who learned about the program), 35.0% (126/360) initiated baseline participation, but only 26.1% (94/360) fully completed the baseline assessment. A total of 81% (76/94) of study participants completed the follow-up assessment.

### Demographics

Demographic data are presented for those who fully completed baseline (ie, *study participants*); results primarily focus on this group of 94 parents. Participants were primarily African American mothers; their preschool-aged children were nearly equally divided between boys and girls. Parents were, on average, 33.56 years old (SD 6.85; for the Consortium group, mean 35.77, SD 6.30; for the Registry group, mean 31.30, SD 6.71). See Table 1 for detailed information about the study participants.

**Table 1:** Demographic characteristics of study participants.

Demographic reported		Overall (N=94), n (%)	Consortium (n=48), n (%)	Registry (n=46), n (%)
**Parent’s relationship to the child**				
	Mother	86 (92)	43 (90)	43 (94)
	Father	3 (3)	3 (6)	0 (0)
	Grandparent	4 (4)	1 (2)	3 (7)
	Choose not to answer	1 (1)	1 (2)	0 (0)
**Parent education**				
	<High school graduate	5 (5)	0 (0.0)	5 (10)
	High school graduate/General Educational Development	28 (30)	8 (17)	20 (45)
	Some college/associate’s degree	27 (29)	10 (20)	17 (37)
	Bachelor’s degree	12 (13)	12 (25)	0 (0.0)
	Advanced degree (master’s degree, doctorate)	19 (20)	16 (33)	3 (7)
	No response	3 (3)	2 (4)	1 (2)
**Child gender**				
	Boy	49 (52)	27 (56)	22 (48)
	Girl	44 (47)	20 (42)	24 (52)
**Child’s age (years)**				
	2	24 (26)	18 (38)	6 (13)
	3	25 (26)	12 (25)	13 (28)
	4	28 (29)	13 (27)	15 (33)
	5	14 (15)	5 (10)	9 (20)
**Race and** **e** **thnicity**				
	African American	68 (72)	27 (56)	41 (89)
	Caucasian	20 (21)	18 (37)	2 (4)
	Hispanic or Latino	0 (0)	0 (0)	0 (0)
	Native American	1 (1)	1 (2)	0 (0)
	Asian	6 (6)	5 (10)	1 (2)
	Middle Eastern	5 (5)	1 (2)	4 (9)
	Other	2 (2)	2 (4)	0 (0)
	Choose not to answer	1 (1)	1 (2)	0 (0)
**Relationship status**				
	Married	36 (38)	22 (46)	14 (30)
	Living with romantic partner	8 (8)	4 (8)	4 (9)
	Never married	40 (43)	13 (31)	27 (59)
	Divorced	7 (7)	6 (13)	1 (2)
	Widow	0 (0)	0 (0)	0 (0)
	Choose not to answer	1 (1)	1 (2)	0 (0)
**Enough money to pay for basic needs**				
	Rarely or never	6 (6)	2 (4)	4 (9)
	Sometimes	9 (10)	2 (4)	7 (15)
	About half the time	14 (15)	3 (6)	11 (24)
	Always	65 (69)	41 (85)	24 (52)

### Participants’ Cumulative Sociocontextual Risk and Use of Five Parenting Activities

**Figure 3:**
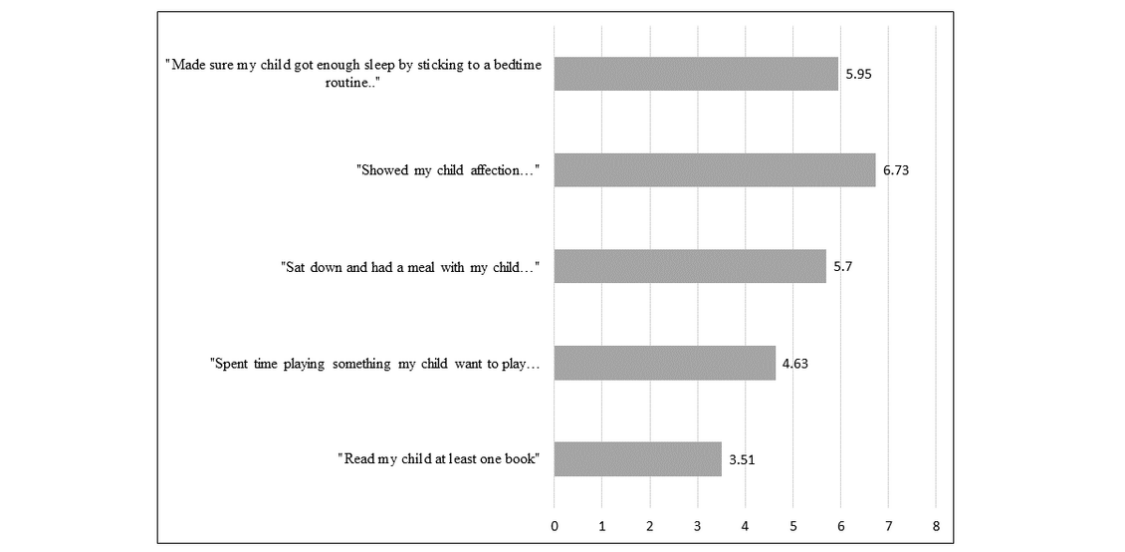
Parent reports of frequency of completing the five parenting activities in the week before participation.

The mean cumulative sociocontextual risk score was 1.33 (SD 1.15). Sociocontextual risk was significantly higher in the Registry group (mean 1.89, SD 1.04) than the Consortium recruitment group (mean 0.79, SD 0.99; *t*_92_=−5.26; *P*=.001). Mean ratings for use of each of the five parenting activities in the week before baseline participation are reported in Figure 3. Reading was the least often reported parenting activity, with 53% of parents reading to their child 3 times or less per week. In contrast, 90% of parents reported expressing affection to their child every day in the previous week. The mean score for the total parenting activities score was 26.23 (SD 6.78); this score was statistically equivalent in the Consortium recruitment group (mean 27.35, SD 6.22) and the Registry recruitment group (mean 25.07, SD 7.20; *t*_92_=7.65; *P*=.10).

### Text Messages

Tracking showed that all study participants received text messages, and only 2 elected to stop receiving messages. At the 4-week follow-up (n=76), 46% (35/76) of parents reported *always* reading the text messages, 21% (16/76) reported reading the text messages *most of the time*, 16% (12/76) reported reading the texts *about half the time*, 9% (7/76) reported reading the texts *sometimes*, 3% (2/76) reported reading them *rarely*, and 5% (4/76) provided no response.

### Website Use

Of the 94 study participants, 33% (31/94) used the website at least once. For study participants who used the website, the average number of visits was 7.1 (SD 10.6). Two parents were identified as outliers, however, as one participant used the website 56 times and one used the website 30 times, in reexamining the data with these outliers removed, mean visits decreased to 4.7 (SD 3.3). Of the parents who used the website, 84% (26/31) used it more than once.

### Recruitment Group, Program-Affiliation Message, Cumulative Sociocontextual Risk, Parenting Activities Frequency, and Website Use

Of the study participants recruited from a Consortium childcare center (ie, face-to-face recruitment), 42% (20/48) used the website at least once, whereas only 24% (11/46) of participants recruited via text message from the registry used the website; this difference was not statistically significant (*χ*^2^_1_ [*N*=94]=3.4, *P*=.07). For the entire sample of baseline participants, program-affiliation message did not affect website use (*χ*^2^_1_ [N=94]=4.7, *P*=.49). When looking at the recruitment group separately, program-affiliation message still did not affect website use (Consortium recruitment group, *χ*^2^_1_ [N=48]=0.5; *P*=.49; registry recruitment group, *χ*^2^_1_ [N=46]=0.4; *P*=.55). There was also no evidence that sociocontextual risk affected parents’ use of the website (*r*=−0.12; *P*=.24). Given that an accumulation of sociocontextual risk was higher in the registry group, the impact of an accumulation of sociocontextual risk on website use was considered separately by recruitment group. An accumulation of sociocontextual risk was not related to website use in either the registry group (*r*=0.11; *P*=.48) or the Consortium recruitment group (*r*=−0.07; *P*=.64). Finally, there was also no association between reported parenting activities at baseline and website use (*r*=0.03; *P*=.90).

#### Parents Ratings of the Text Messages and Website

Table 2 presents ratings from parents on program text messages. When asked if they would sign up for text messages if involved in a project similar to this again, 86% (65/76) reported yes, 8% (6/76) responded no, and 7% (5/76) did not respond. Parents’ feedback on the 5-a-Day Parenting website is presented in Table 3; all 31 parents who participated in the baseline session and used the website completed the 4-week follow-up survey and provided feedback.

**Table 2:** Text message feedback from parents who received text messages and completed follow-up (N=76).

Survey items		Not at all true, n (%)	Somewhat true, n (%)	Very true, n (%)	No response, n (%)
**Positively** **worded items**					
	The text messages were helpful to me.	1 (1)	36 (47)	33 (43)	6 (7)
	The text messages encouraged me to spend time with my child.	10 (13)	32 (42)	39 (51)	6 (7)
	The text messages helped me remember the website.	4 (5)	24 (31)	42 (55)	6 (7)
	The text messages led me to use the website.	4 (5)	33 (43)	34 (45)	5 (6)
	The text messages helped me make parenting changes.	14 (18)	37 (49)	19 (25)	6 (8)
	I liked the text messages.	3 (4)	34 (45)	35 (46)	4 (5)
**Negatively** **worded items**					
	There were too many text messages.	42 (55)	22 (29)	8 (11)	4 (5)
	I stopped reading the text messages after a while.	55 (72)	15 (20)	2 (3)	4 (5)
	The text messages did not change my behavior.	38 (50)	23 (30)	9 (12)	6 (8)

**Table 3:** Satisfaction among participants that used the video-based content on the website (n=31).

Survey items		Not at all True, n (%)	Somewhat true, n (%)	Very true, n (%)	No response, n (%)
**Positively** **worded items**					
	I like the way the website looks.	1 (3)	13 (42)	17 (55)	0 (0)
	It was easy to understand the information on the website.	0 (0)	3 (10)	28 (90)	0 (0)
	It was easy to read through the information on the website.	1 (3)	5 (16)	25 (81)	0 (0)
	I like the videos on the website.	0 (0)	16 (52)	15 (48)	0 (0)
	It was easy to understand the information in the videos.	0 (0)	4 (13)	26 (87)	1 (3)
	I like that there are videos.	1 (3)	5 (17)	24 (80)	1 (3)
	There is lots of good information on the website.	0 (0)	7 (23)	23 (77)	1 (3)
	I found the information on the website to be useful.	0 (0)	9 (29)	22 (71)	0 (0)
	I had an easy time finding things on the website.	2 (7)	12 (39)	17 (55)	0 (0)
	The website is well organized.	1 (3)	12 (39)	18 (58)	0 (0)
	I like the animated characters in the videos.	5 (17)	15 (52)	9 (31)	2 (7)
**Negatively** **worded items**					
	There is too much information on the website.	25 (81)	4 (13)	2 (7)	0 (0)
	There was too much information in the videos.	25 (86)	2 (7)	2 (7)	2 (7)
	The information on the website is confusing.	29 (94)	2 (7)	0 (0)	0 (0)
	I do not like the website.	28 (90)	3 (10)	0 (0)	0 (0)
	I did not find the website helpful.	28 (90)	3 (10)	0 (0)	0 (0)

## Discussion

### Principal Findings and Comparison With Prior Work

This study sought to evaluate use of a light-touch, online parenting program and program satisfaction. This study included 2 enhancements designed to promote use of the program: (1) a brief e-intervention that introduces the program and promotes engagement, and (2) tailored text messages; it further sought to evaluate the extent to which dissemination method, program-affiliation messages, sociocontextual risk, and preintervention use of the parenting strategies might be associated with program uptake.

Among study participants (n=94), approximately a third (ie, 33%) went on to use the 5-a-Day Parenting program website. This rate of website use is lower than reported in other investigations; for example, Doty et al [21], reported that 50% (193/370) of military families used an online parenting program, and Ryan et al [22] reported that 60% (91/158) of parents of children with ADHD used on online parenting resource. However, findings must be interpreted within the context of the sample. Notably, parents in this study were not seeking parenting assistance at the beginning of the project. In addition, no screening for challenges in parenting or child development took place before inviting parents to participate. Although parents who participated in the study were not required to use the 5-a-Day Parenting program website’s video-based content and received no compensation for website use, many chose to use the website and learn positive parenting strategies. Of those who did so, the majority used it more than once, and most rated it as helpful. Moreover, most participants reported reading the messages, and only 2 elected to stop the messages. Only 1 participant responded that the text messages were not helpful, and only 3 reported not liking the messages. These results suggest that a substantial minority of parents, despite not seeking parenting assistance, will use an internet-based parenting program after being invited to do so, which is an encouraging finding.

However, the majority of the 360 parents who learned about the study (74%) chose not to participate. Virtually none of the nonparticipating parents made use of the website (despite having a log-in ID to access the website outside of study participation), suggesting initial engagement (and completion of the brief e-intervention and receipt of text messages) is essential for promoting use of the website content. We do not know enough about this wider group of parents to draw conclusions. There is a need for more research to understand the processes that promote initial engagement in parenting-focused studies and use of parenting resources. Innovative strategies are needed to prompt initial engagement. For instance, providing parents’ space to complete the initial e-intervention of the 5-a-Day Parenting program through services they already use may boost program use. This may look like parents doing the e-intervention at an orientation for childcare or while waiting at a pediatric office. Moreover, direct input from parents may lead to further modifying the program to make it more attractive and appealing to parents. Hansen et al [14] found that gathering suggestions and input from parents (eg, via qualitative methods, such as focus groups), particularly from underserved populations, leads to technology-based parenting programs with higher engagement/retention rates.

We failed to uncover any factors that affect accessing the video-based content on the 5-a-Day Parenting program website. On the basis of the qualitative work of Bernhardt and Felter [24] and Eysenbach and Kohler [25], we expected that program affiliation would affect program use. Specifically, we expected that parents who saw an explicit message about the program creators being experts with an academic/scientific background would be more likely to use the 5-a-Day Parenting program website. However, we found no support to indicate that program affiliation made an impact. Interestingly, Eysenbach and Kohler [25] did note that although people *say* having a scientific source of information is important, they also found that people rarely look for information on websites to investigate the background and training of those who created internet-based resources. This may suggest that program-affiliation may not be crucial in understanding actual use of information on the internet.

Of parents recruited through a Consortium childcare center (ie, face-to-face recruitment), 42% (20/48) used the 5-a-Day Parenting program website; only 24% (11/46) recruited through the registry (ie, via text message) used the website. Although this difference did not reach statistical significance, results may have differed in a larger sample. Further investigation is needed regarding whether differences in recruitment approach (folder vs text) or context (childcare center vs registry) can influence engagement.

There was variability in how often parents reported performing the five parenting activities at baseline. Interestingly, reading was the least often reported activity with approximately half of parents reporting reading to their child less than 3 days in the week before project participation. This is surprising given the importance of book sharing and presence of initiatives to promote it in the preschool years [35]. Expressing affection was the most prevalent activity reported by parents with most parents reporting expressing affection 6 to 7 days in the previous week. Frequency of performing the parenting activities in the previous week was not predictive of using the 5-a-Day Parenting website. This may be because the program was designed to encourage all parents to use the website, even if they already performed the activities frequently. Specifically, for parents who reported regularly performing the activities already, the e-intervention recommended parents use the website to learn how to *make the most* out of the time spent performing the activities (eg, 1 video taught parents how to be responsive and cognitively stimulating in play).

We also did not find associations between cumulative sociocontextual risk and use of the 5-a-Day Parenting program website. Although a nonsignificant finding may be due to multiple factors (eg, low power because of small sample size), the results may also be encouraging. Families facing sociocontextual risk often face practical barriers, such as lack of transportation or childcare, which make participating in face-to-face parenting programs a challenge (see [15]). Web-based parenting programs may be a way for parents facing sociocontextual risk to access the same research-informed parenting information without the challenges of attending face-to-face parenting training. Results of the current investigation echo the findings of Baker et al [15] who reported that high- and low-risk parents are equally open to Web-based parenting resources. In the previous study [23], we also found high openness to internet-based parenting information in a low-income sample. Taken together, it does not appear areas of sociocontextual risk are barriers to online parenting program use. It may be that sociocontextual risk did not deter program because the program is online and Smartphone ownership is high in this population [23], making the program accessible.

### Limitations

There are a number of limitations in this study. First, a clear limitation is the small sample size of the project. Project budget and timeline limited the sample size. The small sample, coupled with the fact that recruitment took place in only 1 US city, limits generalizability of findings. Second, it is unclear why such a sizeable minority of parents (ie, approximately 25% (32/126) of those who started baseline participation did not complete it) initiated, but did not complete, baseline assessment (see Figure 1). Some parents reported technical difficulties in completing the brief e-intervention, but these issues were immediately rectified. However, it is possible that some potential participants experienced technical difficulties but chose to discontinue participation rather than contacting project staff. Third, although parents were not compensated for using the 5-a-Day Parenting website, parents were compensated for baseline participation, which included going through the brief e-intervention and completing the follow-up assessment. Results may have differed without compensation. Finally, it is important to note this investigation does not document impact (ie, efficacy or effectiveness) of the 5-a-Day Parenting program; future investigations are needed to document if program use leads to positive changes in parenting and child outcomes.

### Conclusions

In a general sample of parents who chose to participate in a research project, despite not seeking parenting assistance, approximately one-third of parents made use of internet-based parenting content. Furthermore, those who did use the website rated the program as helpful. These are important findings suggesting that many nontreatment seeking parents may receive benefits through online programs. However, we failed to uncover factors that differentiate those who make use of a Web-based parenting resource and those who do not. Participating parents accepted text messages at a very high rate and viewed them favorably. However, most participants chose not to initially engage in the research project at all, and approximately 70% of those who did engage in the research project did not make use of the website. More research is needed to identify factors predictive of engagement in online parenting interventions, as well as techniques for promoting greater engagement.

## Abbreviations

ADHD: attention-deficit/hyperactivity disorder
